# Nagor Impleo Round Silicone Gel Breast Implants: Early Outcome Analysis after 340 Primary Breast Augmentations

**DOI:** 10.3390/jcm12113708

**Published:** 2023-05-27

**Authors:** Maximilian Zaussinger, Dominik Duscher, Georg M. Huemer

**Affiliations:** 1 Medical Faculty, Johannes Kepler University Linz, Altenbergerstr. 69, 4040 Linz, Austria; 2 Section of Plastic, Aesthetic and Reconstructive Surgery, Kepler University Hospital Linz, Krankenhausstrasse 9, 4020 Linz, Austria; 3 Department of Plastic, Reconstructive, Hand and Burn Surgery, BG-Trauma Center, Eberhard Karls University Tübingen, 72076 Tübingen, Germany; dominikduscher@me.com

**Keywords:** breast surgery, breast augmentation, breast implants, Nagor implants, aesthetic

## Abstract

For decades, implant-based breast augmentation has been one of the most performed surgical procedures for cosmetic purposes around the world. Hence, novel manufactured implants should be critically investigated to prove them safe and effective. Here, the authors describe the first independently conducted clinical study on Nagor Impleo textured round breast implants. For this retrospective study, outcomes of 340 consecutive female patients undergoing primary cosmetic breast augmentation were analyzed. Demographic and surgical data as well as outcomes and complications were evaluated. Furthermore, a survey concerning effectiveness and aesthetic satisfaction after breast augmentation was examined. All 680 implants were placed in a submuscular plane with incisions at the inframammary fold. The main indications for surgery were hypoplasia and hypoplasia with asymmetry. The mean implant volume was 390 cc and the main type of projection was high profile. The most common complications were hematoma and capsular contracture (0.9 percent, respectively). The overall revision rate for complications was 2.4%. Additionally, almost all patients showed increased quality of life and aesthetic satisfaction after a breast augmentation. Hence, all patients would undergo breast augmentation again with these newly launched devices. Nagor Impleo implants demonstrate a low complication rate and high safety profile. Although high aesthetic satisfaction and quality of life results were achieved, analysis of an even larger series over a longer period of time would be beneficial to evaluate the reliability of this implant.

## 1. Introduction

Implant-based breast augmentation is one of the most frequently performed cosmetic surgical procedures worldwide [1,2]. The field has continuously evolved due to various trends in aesthetic breast surgery [3]. Beside surgical considerations such as selection of the pocket plane (submuscular vs. subglandular plane) and incision (inframammary fold vs. periareolar incision), the choice of the right implant is one of the most critical factors for successful breast augmentation. Breast implants have undergone significant changes in shape (anatomical vs. round), surface (smooth vs. textured), and material used for filling (saline vs. silicone) [4]. Since the reapproval of silicone implants by the US Food and Drug Administration in 2004, silicone has become the most commonly used device-fill material due to design and material innovations [5,6]. The latest generation of silicone implants boasts increased structural stability, textured surface, and cohesive gel that retains a natural shape in vivo [7]. However, over recent decades, implant surface, particularly with high texturization, is under suspicion as being associated with anaplastic large cell lymphoma [8]. This entity of malignancy has changed implant selection and manufacturing developments towards less-textured surfaces. On the other side, smooth or nanotextured implant surfaces are predicted to be more scarce due to malpositioning and capsular contracture [9,10,11].

Certified breast implants today come in various shapes, projections, and sizes. Anatomical implants are believed to give a more natural appearance, while round implants increase fullness in the upper pole of the breast [9,10]. However, anatomical implants carry a malrotation risk of up to 5.2% [12]. Ongoing attempts have been made to mimic natural-appearing breasts without postoperative complications, including malposition or rotating [9,10,11].

Nagor Impleo implants, powered by GC Aesthetics (Sandyford, Dublin 18, Ireland), were introduced in 2014 as a solution to obtain a natural look without the risk of rotating. Nagor Impleo implants are currently mainly distributed in Europe and Latin America. The company offers 38 variations (150 cc–620 cc) of round implants with three different types of projection (moderate profile, high profile, and extra-high profile). The base width is available from 8.2 cm to 13.6 cm and Nagor Impleo offers a projection range from 3.4 cm to 6.6 cm. They consist of a round, textured, high-performance silicone elastomer shell filled with a soft, high-cohesive gel. The company claims that the responsive silicone gel system is unbreakable, form-stable, and ergonomic in motion. The newly designed mid-size textured surface promotes natural interaction and minimizes the risk of dislocation, capsular contracture, and late immune response. However, there are no independent clinical data available to prove the safety and effectiveness of these implants. This investigation presents the outcome of 680 Nagor Impleo implants for cosmetic breast augmentation in 340 consecutive patients over 6 years without any company support or input. It analyzes the effectiveness and safety of the implant and provides technical recommendations for incorporating these devices into surgical practice.

## 2. Material and Methods

For this retrospective study, 340 consecutive patients underwent primary cosmetic breast augmentation using Nagor Impleo round breast implants from 2016 to 2022. Written and verbal informed consent was obtained from all patients to publish this paper and the study was conducted following the principles of the Declaration of Helsinki. Formal institutional review board approval was not required according to the retrospective study design. Data were rendered anonymous and stored in a password-secured database on the server of our practice. Basic examinations were performed, and medical history was taken to certify that all patients were in good health. The criteria for exclusion were as follows: patients under 18 years old, pregnant or breastfeeding, with cancer, active fever (temperature >37.5 °C), chronic obstructive pulmonary disease or cystic fibrosis, undergoing radiation therapy or chemotherapy, alcohol or drug abuse, or displaying acute signs of infection. The patient demographics, including age, sex, follow-up, body mass index, and indication, were analyzed. The surgical data, including postoperative complications and effectiveness, were also evaluated.

### 2.1. Surgical Approach

Before undergoing the surgery, the size and projection of the implants were selected based on the patient’s biological characteristics and desired appearance, using external sizers and professional judgment. Preoperative markings were made in a standing position after standardized pictures were taken. After marking the sternal notch and the chest midline, the original inframammary fold and, if necessary, a new defined level of the inframammary fold was determined, which can vary due to individual-related conditions. The procedure was carried out under general anesthesia with the patient in a supine position, and the arms extended at 90° on an arm board. After sterile washing of the patient and the application of nipple shields, incisions were placed at the inframammary fold, and all implants were positioned in a submuscular plane, similar to the dual-plane method [13]. The incision length ranged up to 4 cm, depending on implant size and breast conditions. A subglandular pocket designed like an inverted cone was created by dissecting towards the upper border of the nipple–areola complex (NAC) using monopolar cautery (ValleyLab, ForceFX, Medtronic, Vienna, Austria). The gland was kept attached to the inferior part of the pectoralis major muscle in order to mimic a most natural outcome [14]. The submuscular plane was then dissected by lifting the lateral edge of the pectoralis major muscle until the sternal border. If the width of the pectoralis major muscle exceeds the midclavicular line, it is possible to cut through the muscle. In some cases, the remaining lateral pectoralis major muscle can be used as muscle sling for additional implant stability. Care should be taken to save the pectoralis minor and serratus anterior muscles to minimize bleeding. Our modified dual-plane technique involved separating the muscle from the sternal border at the inferior part of the muscle, which resulted in a better draping of the gland and prevented cranial sliding (window-shading) of the muscle after implant placement [14].

To avoid animation deformity, a median myotomy of the pectoralis major muscle was performed in every dual-plane augmentation, even in young female patients with small breasts who seek aesthetic breast enlargement [15]. In particular, waterfall deformity can be effectively prevented when performing this surgical step. After the implant pocket was made, we required elevated blood pressure (about 120/80 mmHg) to uncover any potential bleedings. Bleedings were cauterized and the breast pocket was flushed with a saline solution with diluted betadine (1:4) prior to implant placement. After changing gloves and disinfecting the incision area, the implant was placed in the newly designed pocket. A final assessment was made to assure the correct, harmonic, and symmetrical appearance of the implants. Following implant placement, the incision was closed in three layers, with multiple soft tissue layers being closed over the implant to minimize the risk of palpability or exposure. Beginning with three-point sutures, the superficial fascial system of the cranial wound edge was fixed to the deep fascia including the superficial fascial system of the caudal wound edge with 3–0 absorbable, interrupted sutures (Polysorb TM; Medtronic, Vienna, Austria). For further wound closure, 3–0 absorbable, interrupted, deep dermal sutures (Polysorb TM; Medtronic, Vienna, Austria) and a running 4–0 intradermal suture (Caprosyn TM; Medtronic, Vienna, Austria) were placed. For this outpatient procedure, we did not use drains. The standard postoperative management included antibiotic prophylaxis, anti-inflammatory therapy, and a compression bra worn continuously for 8 weeks, including a belt on the upper pole for 2 weeks.

Clinical data and postoperative photographs were collected during outpatient checkups after 3 days, 7 days, 14 days, 3 months, 6 months, 1 year, 3 years, and 5 years. 

### 2.2. Survey Analysis

In addition to evaluating demographic and surgical data and postoperative complications, aesthetic satisfaction and quality of life were analyzed using an anonymous Likert-scale questionnaire [16]. The following questions were asked: I would repeat the procedure anytime; my quality of life has improved since breast augmentation; my self-esteem has improved since the breast augmentation; my environment has reacted positively to the breast augmentation; I have no foreign body sensation; I am not limited in sports since the breast augmentation; I recovered fast after the breast augmentation; I am happy with the shape of the breast; and I am happy with the size of the breast. The six-item questionnaire was scored by 3 plastic surgeons and 282 patients at the 1-year follow-up visit and all results were analyzed using descriptive statistics. Statistical analysis was performed using IBM SPSS Version 27.0 (SPSS Inc., Chicago, IL, USA).

## 3. Results

### 3.1. General Analysis

All analyzed patients were women and underwent primary cosmetic bilateral breast augmentation with Nagor Impleo round breast implants. The average follow-up was 38 months (range: 6 to 74 months). The mean age was 31.4 (range: 18 to 61 years) with an average body mass index of 21 kg/m^2^ (range: 17 to 33 kg/m^2^) at date of surgery. Indications for breast augmentation were hypoplasia (66.8 percent), hypoplasia with asymmetry (22.6%), ptosis (7.4%), and hypoplasia with ptosis (3.2%), which are summarized in Table 1. A total of 90% of the ptotic breasts were classified as type 1 (mild breast ptosis) of Regnault’s classification; the other 10 percent were classified as type 2 (moderate breast ptosis) [17]. 

**Figure 1:**
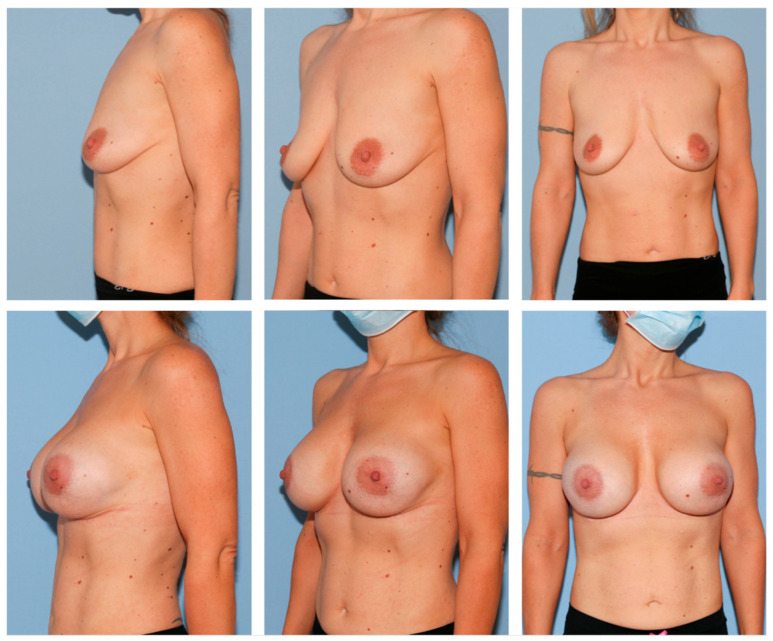
A 35-year-old female patient before (**above**) and 1 year after breast augmentation (extra-high profile, 520 cc, bilateral) (**below**). Note how Nagor Impleo implants can correct ptosis type 2 of Regnault´s classification.

**Figure 2:**
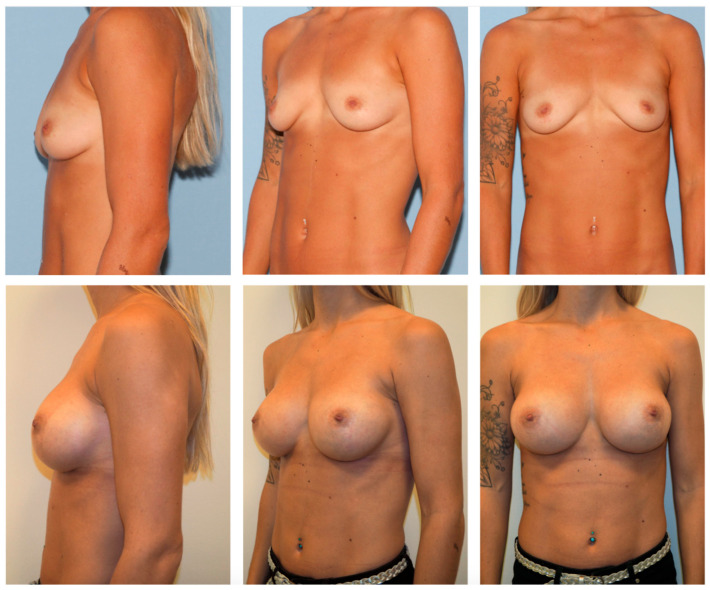
A 27-year-old female patient before (**above**) and 2 years after breast augmentation (moderate profile, 300 cc, bilateral) (**below**). The presenting patient requested a somewhat significant breast augmentation of approximately 2 cup sizes. Although Nagor Impleo has a round shape, the implant follows the body position, mimicking a natural breast appearance.

**Figure 3:**
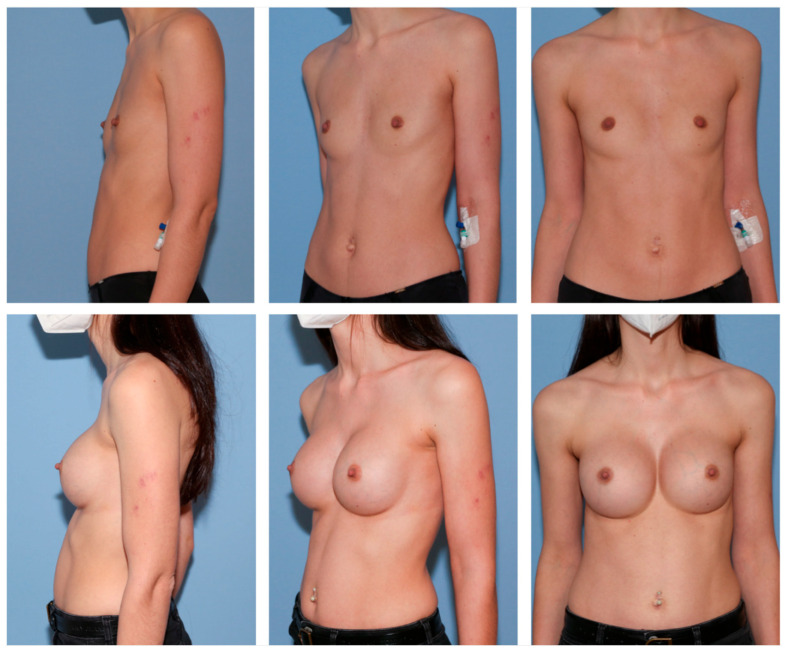
A 22-year-old female patient with breast asymmetry (**above**) and 1 year after breast augmentation (**below**). This patient requested a somewhat significant breast augmentation of approximately 2 cup sizes. To also generate symmetry, we used a high profile 360 cc Nagor Impleo implant for the right breast and a high profile 390 cc Nagor Impleo implant for the left breast.

The mean implant size was 390 cc (range: 240 to 560 cc) and the most commonly chosen projection was high profile (67.5%) followed by extra-high profile (20.1%) (Figure 1) and moderate profile (12.4%) (Figure 2) summarized in Table 2. The most used device (*n* = 190) was the high profile 375 cc implant. The base width of this implant is 11.8 cm, and it has a projection of 5.1 cm. Regarding asymmetry, the average difference between implants was 50 cc (range, 30 to 160 cc) (Figure 3).

### 3.2. Complications

Complications occurred in twelve patients (3.5%). The most common complications were hematoma and capsular contracture which occurred in three patients (0.9%), respectively (Table 3).

In cases of hematoma, evacuation was performed in the first five hours after initial surgery. Concerning capsular contracture, all three patients detected signs of contracture and moderate pain between 24 and 36 months after surgery (Baker grade II/III [18]). A complete capsulectomy was performed and new prostheses were implanted. In one case (0.3%), unilateral implant rupture was sonographically detected after 48 months. After capsulotomy, the ruptured implant was replaced and sent to the manufacturer for investigation. In addition, we observed one (0.3%) implant malpositioning laterally 6 months after surgery. Surgical revision, including implant exchange and plication of the implant pocket, was performed. Further minor complications were successfully treated without surgical intervention and were summarized in Table 3. The overall revision rate for complications was 2.4%. In three patients (0.9%), we performed implant exchange due to aesthetic reasons. All these three patients felt that their augmented breasts were too small. 

### 3.3. Survey Analysis

**Figure 4:**
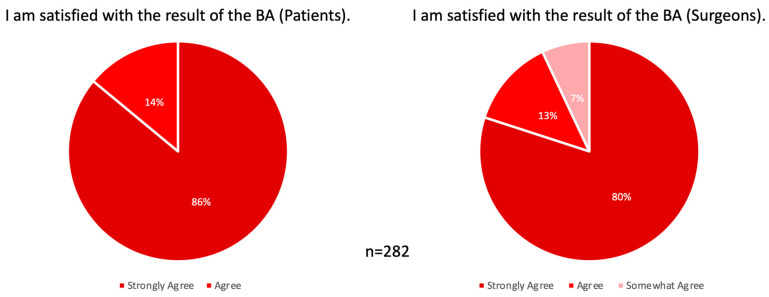
General satisfaction with results 1 year postoperatively. (**Left**) Patients and (**Right**) surgeons showed 100% overall satisfaction with the postoperative results. BA, breast augmentation.

To evaluate satisfaction and quality of life after breast augmentation surgery, a six-item Likert scale questionnaire was conducted 1 year postoperatively [17]. Concerning general satisfaction, 83% of patients strongly agreed and 17% agreed that they were satisfied with their result. In contrast, surgeons felt they strongly agreed with a satisfying result in 80%, agreed in 13%, and somewhat agreed in 7% of cases (Figure 4). 

**Figure 5:**
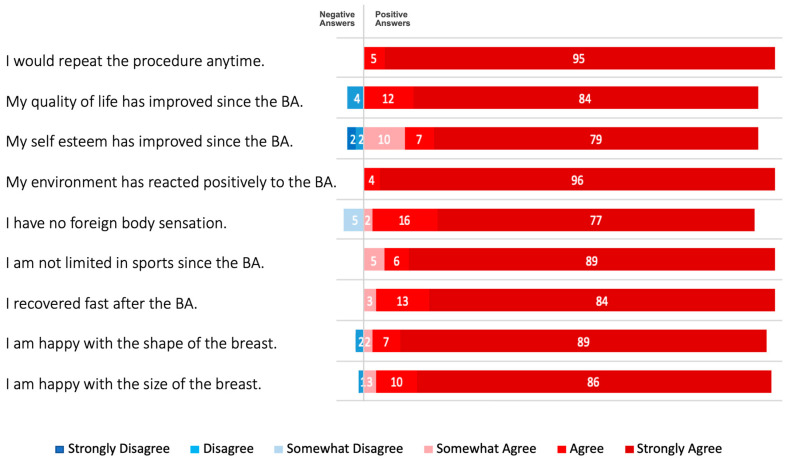
Patient survey outcomes 1 year postoperatively. Breast augmentation with Nagor Impleo implants led to increased quality of life. More than 95% were happy with the size and shape of the breast, respectively. All patients had no foreign body sensation and were not limited in sports since the breast augmentation. In more than 95% of patients, self-esteem had improved and all patients experienced positive reactions from their environment since the breast augmentation. Values are given in percentages (max. 100%). BA, breast augmentation.

Corresponding to satisfaction, 95% of patients strongly agreed and 5% agreed that they would repeat the procedure anytime. Furthermore, 96% of patients strongly agreed and 4% agreed that their environment has reacted positively to the breast augmentation. Regarding quality of life, 4% of patients disagreed that their life quality has improved since the breast augmentation. In contrast, 84% of patients strongly agreed and 12% agreed that their life quality has improved. A total of 1% of patients disagreed that they were happy with the size of the breast and 2% were not satisfied with the shape of their breasts. In contrast, 86% of patients strongly agreed that they were happy with the size of the breast and 89% were very satisfied with the shape of their breasts. A total of 5% of patients disagreed somewhat that they have no foreign body sensation and 4% had no improved self-esteem after breast augmentation, compared to 77% of patients who strongly agreed that they have no foreign body sensation and 79% had highly improved self-esteem after breast augmentation. Further, all patients stated that they recovered fast after the surgery and 89% of patients strongly agreed and 11% somewhat agreed that they were not limited in sports since the breast augmentation. All outcomes are summarized in Figure 5.

## 4. Discussion

The demand for cosmetic breast augmentation surgery continues to grow, requiring consistent development and improvement in the procedure [19]. Consequently, the market for breast implants is more dynamic as well as profit oriented. Hence, newly introduced implants should be critically evaluated in order to ensure patient safety and effectiveness. The recently launched Nagor Impleo round implants must be thoroughly evaluated for these mentioned concerns, as there are no available independent clinical data on their use. This study aimed to examine the surgical outcomes as well as satisfaction after the use of 680 Nagor Impleo round implants for primary breast augmentation.

To achieve an ideal breast appearance after augmentation, surgical technique and implant characteristics must be well-aligned. Technical steps such as management of the inframammary fold or creation of a breast pocket should be considered in implant selection and may affect outcomes. In a previous study on implant-based breast augmentation, we encountered a potential risk of implant malpositioning beyond the inframammary fold, although the prevalence was low (2%) [14]. However, we decided to implement an option in the need of round breast implants. Over a 6-year period, the Nagor implants showed an even lower prevalence of this problem, with only one lateral displacement and no signs of bottoming out. Textured implants are predicted to be more stable and reliable in preventing displacement compared to smooth implants [11,20,21]. The new mid-size textured surface of Nagor Impleo is expected to promote early tissue ingrowth and reduce the risk of malpositioning. Our surgical modification concerning fixation of the inframammary fold (interrupted 3–0 absorbable instead of running 0 nonabsorbable sutures) may also contribute to the stability of these implants within the pocket [14]. In case of lowering the inframammary fold, we recommend to firmly anchor it to the chest wall (periosteum or perichondrium). Besides implant stability, the scar maintains directly in the crease which helps to reduce scar visibility. In general, nipple to inframammary fold distance, the base width of the implant, and individual anatomical conditions such as tissue elasticity and breast parenchyma should be considered in determining the ideal inframammary fold position.

Texturization of the surface may also decrease the risk of capsular contracture and has a lower tendency to develop this pathological immune response than smooth implants [22,23,24]. It is unanimously agreed that a chronic inflammatory reaction after implant-based augmentation leads to this complication. However, the exact pathomechanism of capsular contracture is still unclear [25]. According to the literature, there are several risk factors for capsular contracture, including implant features (smooth shell and smaller size), surgical procedure (periareolar incision, subglandular placement and antibiotic irrigation), postoperative hematoma or seroma, and a longer period of implantation [25,26]. In our patient population, we observed three cases of capsular contracture, which might be related to the use of small size implants (240 cc to 270 cc) [26]. Furthermore, one of these patients evolved hematoma immediately after initial breast surgery. Although proper evacuation and sufficient irrigation was performed before re-implantation, capsular contracture might result out of this adverse event. Even though the incidence of capsular contracture was low, long-term studies are necessary to determine the actual rate for Nagor Impleo implants. There is also an association between shell texturization and the development of breast implant-associated anaplastic large cell lymphoma [27,28,29,30]. In 1997, the first case of anaplastic large cell lymphoma in a patient following breast augmentation (textured saline implant) was reported [31]. Since then, this rare malignancy has changed implant-based breast surgery remarkably. Beside the exponential rise in public interest and the resulting high demand for research, the breast implant industry has adapted their products towards less texturization of implant surface. However, the exact etiology of anaplastic large cell lymphoma is still not fully clarified; thus, newly introduced concepts of textured surfaces should be critical analyzed. In the presenting population, no cases of anaplastic large cell lymphoma or late peri-implant seromas were detected. This absence (at least to date) supports oncological safety, and long-term evaluation to determine the actual incidence is essential. 

A ruptured implant led to revision surgery after four years. Patient examination resulted in overstated forces against the implant. The patient showed no signs of any connective tissue diseases such as fibromyalgia or Raynaud´s syndrome [32]. Surgical revision (capsulotomy and implant exchange), which showed intracapsular rupture, was performed 3 weeks after consultation. Minor complications such as infection or wound dehiscence were treated with prolonged antibiotic therapy and/or specific wound dressings. Compared to similar reports on breast implants, Nagor Impleo implants have low complication rates [33,34].

In terms of effectiveness, our evaluation aligns with previous reports that demonstrate improved quality of life and satisfaction with breast appearance after surgical augmentation [35,36]. Our evaluation also shows that breast augmentation with Nagor Impleo Implants resulted in high patient satisfaction, with positive outcomes on breast shape and foreign body sensation in 95% of cases. This is complemented by high satisfaction with outcomes among the surgeons. Additionally, only 1% of patients were not happy with the size of their breasts. Although size selection is deeply dependent on patient desires and preoperative accordance, a wide range of sizes can be beneficial for the operating surgeon. Our outcomes showed that three patients received exchanges of their implants due to aesthetical reasons. All three patients wished to have notably bigger implants. The versatility of the implant, with a range of projection options, allows for both natural youth appearance and enhanced upper pole fullness. The patients had a fast recovery and were not limited in sports. The ergonomic design of the implant, with a subtle natural nuance even with high projection, received positive reactions from the patients’ environments. As a further result, breast augmentation has improved self-esteem in more than 95% of patients. These positive features might be the reason for all patients in our cohort being willing to repeat the breast augmentation with Nagor Impleo implants.

### Limitations

This study should be seen as a preliminary evaluation of Nagor Impleo implants after cosmetic breast augmentation. While the mean follow-up is comparable to other reports on breast implants, longer-term and larger-scale studies are needed to determine the actual incidence of long-term complications such as anaplastic large cell lymphoma, capsular contracture, or implant rupture [34,37,38]. A well-known general limitation of breast augmentation is severe ptosis. Hence, we would recommend additional mastopexy in such cases. Further potential limitation of the study is that it was conducted at a single center, and the results may not be representative of the general population. However, the results are independent and free from external influence, including the company.

## 5. Conclusions

For the first time, an independent clinical evaluation of Nagor Impleo implants after cosmetic breast augmentation was conducted. All implants were placed in a submuscular plane and incisions were made at the inframammary fold, similar to the dual-plane method. The results showed high levels of safety and effectiveness for both patients and surgeons. Key complications were noted at low rates and no association between the implants and anaplastic large cell lymphoma was found. Due to the high general satisfaction, all patients would undergo breast augmentation with Nagor Impleo implants again. Although we do not consider Nagor Impleo implants to be the sixth generation of implants, their specific characteristics and advanced surgical techniques allow for a variety of desired breast appearances. However, further analysis of a larger patient series over a longer period of time is needed to validate the reliability of our findings. 

## Figures and Tables

**Table 1:** Indications for breast augmentation.

Indication	No. (%)
Hypoplasia	227 (66.8)
Hypoplasia with asymmetry	77 (22.6)
Ptosis	25 (7.4)
Hypoplasia with ptosis	11 (3.2)

**Table 2:** Implant parameters.

Parameter	Value (%)
Overall	680 (100)
Volume, cc	
Mean	390
Range	240–560
Projection	
Extra-high	137 (20.1)
High	459 (67.5)
Moderate	84 (12.4)
Asymmetry	
Mean difference, cc	50
Range	30–160

**Table 3:** Complications and Revisions.

Complication	Prevalence (%)	Surgical Revision
Overall	12 (3.5)	8 (2.4)
Hematoma	3 (0.9)	3 (0.9)
Capsular contracture	3 (0.9)	3 (0.9)
Implant rupture	1 (0.3)	1 (0.3)
Implant malposition	1 (0.3)	1 (0.3)
Infection	2 (0.6)	-
Wound dehiscence	2 (0.6)	-

## Data Availability

Data is contained within the article.

## Abbreviations

NAC—nipple–areola complex.
